# Can leisure education effect on screen time and perceived leisure benefits for college students?

**DOI:** 10.3389/fpsyg.2024.1477857

**Published:** 2024-11-21

**Authors:** Abdullah Bora Özkara, Olcay Mulazimoglu, Mustafa Baris Somoglu, Fatih Kirkbir, Erdi Tokul, Burakhan Aydemir, Halil Evren Senturk, Ibrahim Dalbudak, Ozgun Mirac Ozcilingir

**Affiliations:** ^1^Faculty of Health Sciences, Karadeniz Technical University, Trabzon, Türkiye; ^2^Faculty of Sports Sciences, Mugla Sitki Kocman University, Muğla, Türkiye; ^3^School of Physical Education and Sport, Gümüşhane University, Gümüshane, Türkiye; ^4^Faculty of Sports Sciences, Avrasya University, Trabzon, Türkiye; ^5^Faculty of Sports Sciences, Uşak University, Uşak, Türkiye; ^6^Faculty of Sports Sciences, İstanbul Rumeli University, Istanbul, Türkiye

**Keywords:** physical education, leisure activities, screen time, college students, exercise and screen time

## Abstract

**Background:**

Since children and adolescents usually spend their theoretical course time at schools, they experience many negative effects of inactivity and physical inactivity. They are disadvantaged in terms of facing many health and social problems due to lack of physical activity. This sedentary life increases their exposure to screens and the time they spend sitting.

**Aim of the study:**

The first aim of this research is to conduct an experimental study to reduce the excessive screen time of college students with an exercise intervention. The second aim of the study is to examine the perceived benefits of leisure activities of students with an exercise intervention.

**Methods:**

In this direction, the study group of the research, which was conducted as an experimental study, consisted of 176 [74 female (42.0%), 102 male (58.0%)] students studying in the department of mathematics at college. In the study, the screen exposure time of students was measured with the “Screen time scale for youth and adolescents” along with a personal information form. In addition, the “Perceived Leisure Benefit Scale” scale was used to evaluate the participants’ awareness of the benefits obtained from leisure activities.

**Results and conclusion:**

According to the findings of the research, it was determined that there was a positive and significant decrease in the screen time of the experimental group participants of students who participated in the research before and after the exercise intervention. In the Leisure Benefit Scale, it was observed that there were significant positive differences in favor of the experimental group.

## 1 Introduction

The increased use of technology and screens in our daily lives has raised concerns about the negative effects it may have on our physical and mental well-being. In today’s digital age, screen time has become a pervasive aspect of daily life for individuals of all ages. Whether it is for work, leisure, or education, screens have become an integral part of our lives.

Research has shown that excessive screen time, which includes activities such as watching television, playing video games, and using computers and digital devices, can have negative effects on cognition and mental health, particularly for children, adolescents, and young adults (Neophytou et al., 2021). Numerous studies have highlighted the detrimental effects of excessive screen time on cognitive development, including learning and memory (Mineshita et al., 2021; Lissak, 2018; Domingues-Montanari, 2017; Stiglic and Viner, 2019; Radesky and Christakis, 2016; Eric, 2021; Twenge and Campbell, 2018; Li et al., 2020; Muppalla et al., 2023; Must and Tybor, 2005; Hawi and Samaha, 2016; Rahimah, 2021). In response to these concerns, there has been a growing interest in implementing exercise interventions to counteract the negative effects of excessive screen time, especially among students. Physical activity has been widely recognized for its positive impact on cognitive functions, including attention, memory, and information processing (Donnelly et al., 2016; Bidzan-Bluma and Lipowska, 2018; Erickson et al., 2019).

When discussing the physical, psychological, sociological and economic benefits of leisure, the concept of an “improved condition” comes to the fore. One of the important ways to do this is to evaluate physical activities (running, cycling, swimming, etc.; Driver et al., 1991). Leisure Benefits are evaluated as the subjective perception of individuals after they participate in various activities in their free time to improve their personal conditions and meet their individual demands (Li et al., 2021). Therefore, these perceptions may differ among individuals. In previous studies, leisure benefits have been evaluated with various aspects such as physical, physiological, social, educational, esthetic and relaxation (Akgül et al., 2018). Ho (2008) used the Leisure Benefits Scale (LBS) as a reference to measure the leisure benefits of subjects in terms of physical, psychological and social aspects. The Physical Aspect measures the physical benefits (such as disease prevention and control) that an individual derives from participation in leisure and sports activities; the psychological aspect measures the individual’s psychological benefits (such as personal growth and reduction of mental stress), and the social aspect measures the individual’s social benefits (such as the development of social relationships and family ties).

By incorporating regular exercise into the daily routine of students in the mathematics department, it is possible to mitigate the potential social, health and cognitive challenges associated with excessive screen time.

In Turkey, mathematics students are at a disadvantage in terms of the opportunity to exercise regularly due to the sporting competencies of their faculties. Since there are no sports instructors in their departments, they need external support. The most dangerous thing for this group who are far away from sports and exercise is to start and quit sports. Because they may think that sports are not suitable for them and that they cannot have fun. For this reason, the exercise intervention implemented in this study was planned to be fun, not competitive. The leisure activities can be preferred because of the predominance of the fun aspect when starting sports. Students are directed to have fun and move enough, not to be compared in terms of success. The aim of this orientation is to increase the students’ belief in the benefits they will gain from leisure activities when the intervention program is over. Thus, the possibility of students to include leisure sports as an exercise in their individual lives can be increased (Brown and Smith, 2018; Smith, 2019; Zaytsev et al., 2013). The exercise intervention determined in this respect was applied to the students as a regular leisure education. For 3 days a week, volleyball, basketball and futsal leisure activities were added to the weekly programs of the students with 2-h lessons. With this planning, it was aimed to overcome the lack of physical activity in students’ daily lives to a certain extent. When we look at the university youth, it is seen that screen time increases not only in their social lives but also in their academic lives. Nowadays, one of the important areas where technology, in other words screens, are used is mathematics education. Computer and mobile technology have been incorporated into math classrooms to enhance learning experiences and improve student outcomes. However, the excessive use of screens and digital devices, including smartphones, tablets, and computers, has raised concerns about its potential negative impact on students’ health and academic performance (Lissak, 2018; Sanz-Martín et al., 2022; Oswald et al., 2020; Adelantado-Renau et al., 2019; Hale and Guan, 2015; Twenge et al., 2018; Lauricella et al., 2015; Schmidt et al., 2020; Twenge et al., 2018; Tandon et al., 2012; Madigan et al., 2019; Maras et al., 2015; Lanningham-Foster et al., 2006; Davies et al., 2012). The increase in screen time not only for children and young students, but also for adults and especially the elderly negatively affects their quality of life (Davies et al., 2012; Nguyen et al., 2020; Schoeppe et al., 2016; Barr-Anderson et al., 2021; Guo et al., 2021; Colley and Saunders, 2023; Zhang et al., 2021; Ren et al., 2023; Lopes et al., 2023; Hsueh et al., 2018). In the studies conducted by Zhang et al. (2021) important findings were made regarding screen time and electronic device use, especially before sleep. The frequent use of alcohol by the person was found to be an important factor that increased both the time spent in front of the screen and the use of electronic devices before sleep. In addition to this study, Ren et al. (2023) also found in their study that long time spent in front of the screen is associated with an increased risk of stroke, which is more pronounced in men, and that the time spent in front of the screen should be limited to improve health. Hsueh et al. (2018) stated in their study that interaction with the environment is a very important factor for free-time physical activity and screen time. These findings suggest that policymakers and physical activity intervention designers need to develop both joint and individual environmental strategies to improve and increase awareness of the social environment to promote leisure-time physical activity and reduce screen time among older adults.

With the introduction of “screens” into our lives, peers and activities with peers have begun to be neglected and physical and mental health has begun to deteriorate. It is recognized that screen time is linked to sedentary behavior, especially among young people, and contributes to the increase in obesity, which is considered a risk factor in global mortality (Lissak, 2018; Domingues-Montanari, 2017; Li et al., 2020; Muppalla et al., 2023; Brzęk et al., 2021; Nakshine et al., 2022; Khouja et al., 2019; Abbasi et al., 2021; Ratan et al., 2020). Leisure activities, including physical, cognitive and social activities, are an important component of an individual’s return to a healthy lifestyle. However, young people need to be supported in perceiving the benefits they can obtain from these activities. In this study, the perceived benefit level of students toward leisure activities preferred as exercise intervention was also examined. Thus, in order to increase the permanence of reducing screen time in mathematics students, it was aimed to continue their awareness after the program. Because physical activity should be a lifelong sustainable behavior rather than taking place in only one period of young people’s lives. Mathematics department students, on the other hand, are distanced from exercise both in their university life and in their professional life after university because they usually work at a desk and are exposed to the screen. Although education and working conditions are responsible for this distancing, it is also important that they are aware of the benefits they will get from leisure activities, which are preferred as exercise intervention in this research. For this reason, the first aim of this research is to reduce the excessive screen time of students continuing their education in the mathematics departments of universities with an exercise intervention, while the second aim is to examine the benefits that mathematics department students perceive from leisure activities with an exercise intervention.

## 2 Methods

### 2.1 Participants

The sample of this study consisted of 176 volunteer university students (58% female, 42% male) studying in mathematics departments at universities in Trabzon province in the 2023–2024 academic year. The demographic information of the participants is presented in the table below with their percentages.

The Table 1 shows the demographic characteristics of the study group and the distribution between the groups. The experimental group (EG) represented 48.3% of the participants, while the CG (CG) represented 51.7%. The proportion of female participants was 42.0% and the proportion of male participants was 58.0%. 57.4% of the participants were aged 21 years and younger and 42.6% of the participants were aged 22 years and older. When the Screen Viewing Time (SVT) of the participants is analyzed, 35.8% of the participants spent between 1 and 2 h looking at the screen, while 64.2% spent more than 2 h looking at the screen. It is observed that most participants look at the screen for more than 2 h.

**Table 1:** Demographic data.

Variables	Group	N	%
Study group	EG	85	48.3
	CG	91	51.7
Gender	Female	74	42.0
	Male	102	58.0
Age	≤ 21 years	101	57.4
	>21 years	75	42.6
Screen viewing time	1–2 h	63	35.8
	2 h+	113	64.2

### 2.2 Data collection scales and procedures

***The Personal Information Form***: This form includes the variables of gender, age and screen time of the participants. According to the data obtained from these variables, measurement tools such as screen time and leisure benefit scale were analyzed.

***Screen Viewing Time (SVT)***: Based on the screen time limits set by the Canadian Pediatric Society, this scale records the responses to the question “How much time did you spend watching TV, Netflix, YouTube, Xbox, streaming video or anything else after school last week?.” The scale includes the options “I have never done this activity,” “more than 30 min,” “30 min to 1 h,” “1–2 h,” and “2 h or more” (Canadian Paediatric Society, 2017). We scored the answers between 1 and 5 in order to analyze the screen time of mathematics students. A score of 1 corresponds to the answer that they are never exposed to the screen, while a score of 5 means that they spend more than 2 h a day on the screen. According to the Canadian Pediatric Society, a limit of 2 h per day has been set.

***Leisure Benefit Scale (LBS)***: In order to measure leisure benefit levels, “The Leisure Benefits Scale,” which was prepared and developed by Ho (2008) and whose Turkish adaptation, validity and reliability was carried out by Akgül et al. (2018) was applied. The LBS is a 5-point Likert-type scale and consists of 24 items and 3 factors. The rating is made as “Strongly disagree = 1, Strongly agree = 5.” The Cronbach Alpha value of the scale is 0.83 and the internal consistency coefficients for the three sub-dimensions vary between 0.80–0.86. To determine the physical benefits perceived by the participants through the LBS, there are topics such as improving cardiopulmonary and physical fitness, protecting from diseases, having a good body shape, contributing to body development, helping to release energy, improving sleep quality, eliminating lethargy caused by daily work and helping to renew energy. To determine the psychological benefits, there are items such as relieve mental stress, to obtain a pleasurable mood, confirm his/her ability by leisure or sport activities, to develop his/her potential abilities, to be more satisfied with his/her life or work, cultivate an active personality to face challenges, to cultivate an independent personality, enjoy and learn new experiences and knowledge. To determine the social benefits, there are items such as improve relationships with my friends or peers, improve family harmony, establish the concept of teamwork, develop his/her social relationship and make new friends, trust from other participants in an activity, support from other participants in an activity understand the different feelings of other participants, share my opinion and thoughts with other participants, to improve my relationship with family and friends (Akgül et al., 2018).

***Leisure Activities***: The content of the exercise intervention determined to be applied to the EG of mathematics department university students was recreational. With the participation of all students, 3 different activities were organized in the gym for 2 h each 3 days a week. Rest was given the day after each activity. The exercise intervention was applied as basketball on Monday, Futsal on Wednesday and Volleyball on Friday. The attendance of the participants was recorded and 16 students who were absent for more than 2 weeks were excluded from the study.

### 2.3 Statistical analysis

The data analysis process with SPSS 25.0 was first evaluated using Kolmogorov–Smirnov tests to test whether normality was achieved. However, the results of these tests did not provide normality analysis. In this case, kurtosis and skewness values, another parameter of the normality indicator, were evaluated. According to Tabachnick et al. (2013), kurtosis and skewness values between −1.5 and + 1.5, which is an acceptable range for analysis, are suitable for parametric testing. Upon the failure to ensure normality, other parametric tests were analyzed. In this study, descriptive statistics, reliability analysis, t-test for independent groups, and parametric tests for repeated measurements (Paired Sample T-test) were used. These analyses allow for deepening and interpretation of the analyses depending on the characteristics of the data set and the characteristics of the research questions. In the study, *p* < 0.05 was accepted as a criterion as an indicator of the significance level of data analysis. In the present study, Cohen’s d values were used to measure the effect size in statistical analyses. In the interpretation of Cohen’s d values, a value of 0.2 or less indicates a small effect size (S), 0.5 indicates a medium effect size (M), and 0.8 or more indicates a large effect size (L) (Cohen, 1988).

## 3 Results

Table 2 shows the arithmetic mean, standard deviation, kurtosis and skewness values of the participants’ total and sub-dimensions of the LBS pre-test and post-test scores. When the kurtosis and skewness values of the pre-test and post-test scores of the total and sub-dimensions of the LBS of the participants were examined, it was determined that the data showed a normal distribution with kurtosis and skewness values between +1.5 and −1.5 and the data were suitable for analysis. When the arithmetic mean scores were examined, it was seen that the participants exhibited LBS scores above the average (Table 2).

**Table 2:** LBS scale pre-test and post-test score descriptive statistics.

Test	Scales	No. of items	N	Min	Max	X	*sd*	Skewness	Kurtosis	Kol.-Smi.	Sha.-Wilk
Pre-test	LBS	24	176	2.75	4.13	3.69	0.32	−0.91	0.25	0.00	0.00
	Physical	8	176	2.50	4.38	3.48	0.35	0.01	0.70	0.00	0.00
	Psychological	8	176	2.25	4.63	3.65	0.47	−1.04	1.23	0.00	0.00
	Social	8	176	2.38	5.63	4.00	0.59	−0.55	0.86	0.00	0.00
Post-test	LBS	24	176	1.71	5.00	3.90	0.64	−0.47	0.40	0.00	0.00
	Physical	8	176	1.75	5.00	4.01	0.67	−0.69	0.70	0.00	0.00
	Psychological	8	176	2.38	5.63	4.19	0.81	−0.14	−0.58	0.03	0.01
	Social	8	176	2.50	4.50	3.61	0.50	0.15	−0.98	0.00	0.00

According to the results of the t-test for independent groups applied to determine whether the participants’ LBS pre-test scores were at a similar level between the groups (EG/CG), it was found that there was no significant difference between the EG and CG [t_(174)_ = 0.25, *p* > 0.05], (Table 3). In other words, it can be said that both the EG and CG participants who participated in the study had similar LBS scores.

**Table 3:** Pre-test comparison of the participants.

Scales	Group	n	*X*	SD	df	t	*p*
LBS	EG	85	3.70	0.31	174	0.25	0.804
	CG	91	3.69	0.34			

In Table 4, the difference in the LBS scores of the individuals participating in the study was examined by t-test analysis applied for repeated measurements before and after exercise. There is a statistically significant difference between the LBS scores of the individuals participating in the research before and after the leisure activity and it is seen that the post-test scores have changed compared to the pre-test scores and the post-test scores have increased (*t* = −14.88, *p* < 0.01). When the sub-dimensions were examined, it was determined that the pre-test and post-test scores differed significantly in the physical (*t* = −7.14, *p* < 0.01), psychological (*t* = −11.81, *p* < 0.01) and social (*t* = −10.67, *p* < 0.01) dimensions, and there was an increase in the post-test LBS scores. When the LBS scores of the participants in the CG before and after the leisure activity were examined, it was seen that there was a statistically significant difference between the LBS scores and the post-test scores changed compared to the pre-test scores and the LBS scores decreased (*t* = 6.57, *p* < 0.01). In the context of sub-dimensions, it was found that the pre-test and post-test scores of the CG differed significantly in the physical (*t* = 5.37, *p* < 0.01) and social (*t* = 8.47, *p* < 0.01) dimensions, while no significant difference was found in the psychological (*t* = 1.27, *p* > 0.05) dimension. When the significant difference was analyzed, a decrease was observed in the post-test scores of the individuals in the physical and social dimensions. In other words, it can be said that the subject group increased their LBS scores by participating in leisure activities, while the CG decreased their LBS scores by not participating in leisure activities. After the Cohen’s d analysis applied to determine the effect level of the significant difference, it was seen that the effect dimension was high in the subject group and medium in the CG.

**Table 4:** Comparison for LBS pre-test and post-test scores before and after the activity.

Group	Scale	Test	X	SD	X difference	SD difference	t	*p*	Effect size
EG (n:85)	LBS	Pre-test	3.70	0.31	−0.69	0.43	−14.88	0.001**	2.02 (L)
		Post-test	4.39	0.37					
	Physical	Pre-test	3.55	0.37	−0.41	0.53	−7.14	0.001**	1.05 (L)
		Post-test	3.96	0.41					
	Psychological	Pre-test	3.67	0.48	−0.79	0.62	−11.81	0.001**	1.77 (L)
		Post-test	4.46	0.41					
	Social	Pre-test	3.94	0.54	−0.81	0.70	−10.67	0.001**	1.38 (L)
		Post-test	4.74	0.62					
CG (n:91)	LBS	Pre-test	3.69	0.34	0.25	0.36	6.57	0.001**	0.60 (L)
		Post-test	3.44	0.48					
	Physical	Pre-test	3.41	0.33	0.13	0.24	5.37	0.001**	0.40 (M)
		Post-test	3.28	0.31					
	Psychological	Pre-test	3.63	0.47	0.04	0.33	1.27	0.207	null
		Post-test	3.58	0.59					
	Social	Pre-test	4.06	0.63	0.39	0.44	8.47	0.001**	0.63 (L)
		Post-test	3.67	0.60					

**p < 0.01 the significance level.

The results of the t-test for repeated measurements applied according to the comparison results of the pre-test and post-test scores of the male and female participants in the EG and the CG are presented in Table 5. According to the results of this comparison, a significant difference was observed in the pre-test and post-test scores of the female and male participants in the subject group after the leisure activity, and a significant increase was observed in the post-test LBS total and sub-dimension scores (*p* < 0.01). When the pre-test and post-test scores of the CG female and male participants were examined, it was determined that there was a significant difference in the total and physical and social sub-dimensions of the LBS and the post-test scores decreased (*p* < 0.05), while the LBS scores did not change in the psychological dimension (*p* > 0.05). After Cohen’s d analysis was applied to determine the effect size, it was seen that the effect size was strong in the subject group and moderate in the CG.

**Table 5:** Comparison of LBS pre-test and post-test scores before and after the activity according to gender.

Gender	Group	Scale	Test	X	SD	X diff.	SD diff.	t	*p*	Effect size
Female	EG (n:37)	LBS	Pre-test	3.67	0.32	−0.69	0.43	−9.80	0.000**	1.88 (L)
			Post-test	4.36	0.41					
		Physical	Pre-test	3.55	0.37	−0.41	0.50	−4.96	0.000**	1.11 (L)
			Post-test	3.96	0.37					
		Psychological	Pre-test	3.68	0.50	−0.75	0.57	−7.94	0.000**	1.69 (L)
			Post-test	4.43	0.38					
		Social	Pre-test	3.86	0.52	−0.83	0.73	−6.88	0.000**	1.32 (L)
			Post-test	4.69	0.72					
	CG (n:37)	LBS	Pre-test	3.63	0.37	0.25	0.41	3.70	0.000**	0.49 (M)
			Post-test	3.38	0.62					
		Physical	Pre-test	3.28	0.34	0.07	0.22	2.06	0.049*	0.25 (S)
			Post-test	3.20	0.30					
		Psychological	Pre-test	3.53	0.54	0.02	0.36	0.28	0.780	null
			Post-test	3.51	0.71					
		Social	Pre-test	4.02	0.68	0.24	0.34	4.30	0.000**	0.73 (M)
			Post-test	3.78	0.67					
Male	EG (n:48)	LBS	Pre-test	3.72	0.31	−0.69	0.43	−11.08	0.000**	2.16 (L)
			Post-test	4.41	0.33					
		Physical	Pre-test	3.55	0.37	−0.42	0.56	−5.13	0.000**	1.00 (L)
			Post-test	3.96	0.44					
		Psychological	Pre-test	3.67	0.46	−0.82	0.65	−8.72	0.000**	1.84 (L)
			Post-test	4.49	0.43					
		Social	Pre-test	3.99	0.56	−0.79	0.67	−8.09	0.000**	1.45 (L)
			Post-test	4.78	0.53					
	CG (n:54)	LBS	Pre-test	3.72	0.31	0.25	0.33	5.56	0.000**	0.71 (M)
			Post-test	3.48	0.36					
		Physical	Pre-test	3.50	0.29	0.17	0.24	5.28	0.000**	0.58 (M)
			Post-test	3.33	0.30					
		Psychological	Pre-test	3.69	0.40	0.06	0.31	1.50	0.140	null
			Post-test	3.63	0.50					
		Social	Pre-test	4.09	0.60	0.49	0.47	7.66	0.000**	0.85 (L)
			Post-test	3.60	0.54					

*p < 0.05 and **p < 0.01 the significance level.

The t-test results for independent groups applied according to the pre-test and post-test score differences of the male and female participants in the EG and the CG are shown in Table 6. According to these results, no significant gender-related difference was detected in the pre-test and post-test score differences of the male and female participants in the subject group after the leisure activity (*p* > 0.05). When the pre-test and test score differences of the male and female participants in the CG were examined, it was determined that while there was no significant difference in the total, physical and psychological dimensions of the LBS (*p* > 0.05), the scores of the LBS changed in the social dimension (*p* < 0.05). It was found that this significant difference was due to the decrease in the post-test LBS scores of men compared to women in physical and social dimensions. When Cohen’s d result was examined, it was determined that the significant effect was at a medium level.

**Table 6:** Comparison of LBS pre-test and post-test scores before and after the activity according to gender.

Group	Scale	Gender	n	X	SD	df	t	*p*	Effect size
EG (n:85)	LBS	Female	37	0.69	0.43	83	−0.06	0.949	null
		Male	48	0.69	0.43				
	Physical	Female	37	0.41	0.50	83	−0.10	0.924	null
		Male	48	0.42	0.56				
	Psychological	Female	37	0.75	0.57	83	−0.50	0.618	null
		Male	48	0.82	0.65				
	Social	Female	37	0.90	0.78	83	0.58	0.565	null
		Male	48	0.81	0.73				
CG (n:91)	LBS	Female	37	−0.25	0.41	89	−0.02	0.988	null
		Male	54	−0.25	0.33				
	Physical	Female	37	−0.07	0.22	89	1.97	0.052	null
		Male	54	−0.17	0.24				
	Psychological	Female	37	−0.02	0.36	89	0.65	0.520	null
		Male	54	−0.06	0.31				
	Social	Female	37	−0.24	0.34	89	2.57	0.012*	0.52 (M)
		Male	54	−0.46	0.49				

*p<0.05 the significance level.

The results of the t-test for independent groups applied according to the pre-test and post-test score differences of the participants in the subject and CGs who participated in the EG depending on the age variable are shown in Table 7. According to these results, no significant difference was found in the pre-test and post-test score differences of the participants in the subject and CGs after the leisure activity due to age (*p* > 0.05).

**Table 7:** Comparison of LBS pre-test and post-test scores before and after the activity according to age.

Group	Scale	Age	n	X	SD	df	t	*p*
EG (n:85)	LBS	≤ 21	48	0.63	0.44	83	−1.40	0.167
		> 21	37	0.76	0.40			
	Physical	≤ 21	48	0.35	0.54	83	−1.24	0.217
		> 21	37	0.49	0.52			
	Psychological	≤ 21	48	0.71	0.62	83	−1.28	0.204
		> 21	37	0.89	0.61			
	Social	≤ 21	48	0.84	0.79	83	−0.10	0.918
		> 21	37	0.86	0.69			
CG (n:91)	LBS	≤ 21	53	−0.27	0.43	89	−0.65	0.515
		> 21	38	−0.22	0.23			
	Physical	≤ 21	53	−0.12	0.25	89	0.67	0.506
		> 21	38	−0.15	0.22			
	Psychologial	≤ 21	53	−0.08	0.33	89	−1.16	0.250
		> 21	38	0.00	0.32			
	Social	≤ 21	53	−0.41	0.44	89	−1.02	0.310
		> 21	38	−0.32	0.46			

*p<0.05 the significance level.

In Table 8, the difference in the screen viewing variable scores of the individuals participating in the study was examined with the t-test analysis applied for repeated measurements before and after exercise. A statistically significant difference was detected between the SVT scores of the EG before and after the leisure activity [t_(84)_ = 7.32, *p* < 0.01]. This significant difference indicates that the time spent looking at the screen by the participants in the subject group decreased before and after exercise. When the effect size impact is examined, it is seen that the effect size of the significant difference is high. When the SVT of the CG was examined, no significant difference was detected [t_(90)_ = −1.52, *p* > 0.05].

**Table 8:** Comparison of SVT scores before and after the activity.

Group	SVT test	X	SD	X (diff.)	sd (diff.)	t	*p*	Effect size
EG (n:85)	Pre-test	1.55	0.50	0.41	0.52	7.32	0.000**	0.95 (L)
	Post-test	1.14	0.35					
CG (n:91)	Pre-test	1.73	0.45	−0.05	0.35	−1.52	0.132	null
	Post-test	1.78	0.42					

**p < 0.01 the significance level.

In Table 9, it was examined whether the difference scores of the individuals participating in the study in the scores of the variable of looking at the screen before and after the exercise created a significant difference according to gender with one-way ANOVA analysis. There was a statistically significant difference between the scores of looking at the screen in the EG before and after the leisure activity in both females [F_(1–72)_ = 20.56, *p* < 0.01] and males [F_(1–102)_ = 20.29, *p* < 0.01]. This significant difference indicates that the time spent looking at the screen decreased in both male and female participants before and after the exercise. When the effect size of the significant difference was analyzed, it was concluded that the effect size was strong.

**Table 9:** Comparison of pre-screening and post screening times of the study group according to gender.

SVT	Group	n	X	SD	df	F	*p*	Effect size
Female (n:74)	EG	37	−0.46	0.56	1–72	20.56	0.000**	1.05 (L)
	CG	37	0.05	0.40				
Male (n:102)	EG	48	−0.38	0.49	1–100	29.29	0.000**	1.08 (L)
	CG	54	0.06	0.30				

**p < 0.01 the significance level.

## 4 Discussion

When we evaluate the results of the EG and CG participants, we see that there is a positive change in the first aim of our research, which is to reduce the excessive screen time of students continuing their education in the mathematics departments of universities with an exercise intervention. We see that there is a significant decrease in the screen time averages of the EG in the post-test data compared to the pre-test data. It was revealed that the EG participating in leisure activities decreased their screen time, while the CG not participating in leisure activities did not have a significant change in the screen time variable and their habits continued. From these results, it can be emphasized that exercise intervention is effective on screen time.

The second aim of our research, which is the benefit perceived by the mathematics department students participating in leisure activities, changed positively as a result of the examination of the RBF. In the EG, a significant increase was observed in the mean scores of the students who participated in leisure activities before and after the activity, while in the CG, which did not participate in the activities, a significant decrease was observed in the mean scores of the LBS in the opposite direction. The significant upward trend in all three of the physical, psychological and social benefit dimensions of the EG, which are the sub-dimensions of the LBS, shows that recreational activities transform the screen time habits of the person into physical, psychological and social benefits with physical activity activities.

Although the LBS scores of both groups before the recreational activities were above the average and there was no significant difference between them. The increase in the mean scores of the EG after the activities, the decrease in the CG and the significant difference between them reveal the usefulness of the activities. These results suggest that leisure programs have positive effects on participants and that these programs can provide beneficial results.

In the study conducted by Karaküçük et al. (2019) on orienteering athletes, it was stated that the RBF scores were at a medium level, the highest benefit score was in the physical dimension and the lowest benefit sub-dimension score was in the psychological dimension. It was stated that leisure activities provide very good physical, psychological and social benefits to adult individuals (Kurkcu Akgonul et al., 2023). Participation in leisure time activities is among the characteristics of social acceptance and the feeling of being able to communicate with each other more comfortably and more pleasantly, to relate equally and to be included in a group (Devine, 2004).

According to the findings of our study, we see that the biggest difference between the pre-test and post-test LBS score averages of the EG occurred in the social benefit sub-dimension. These results can be evaluated as the fact that team sports (basketball, volleyball, futsal) activities among the leisure activity types have a higher effect on socializing the person. When we look at the social benefit dimension, we see that the relationships of the EG participants with friends, peers and family members developed and improved after the leisure activities. The increase in the formation of the concept of teamwork, the development of social relationships and making new friends, the increase in the sense of trust toward others, the increase in the sense of receiving support from others and understanding the other person, the increase in sharing opinions and thoughts with others have emerged as important results in terms of social benefits. In fact, the EG participants gained an increase in their social aspects while reducing their screen time thanks to the leisure activities. This situation may have increased the level of self-awareness and psychological benefits. In fact, as a result of the LBS pre-test and post-test measurements in the EG, the sub-dimension with the highest change effect size was psychological benefits. The development of the participants’ relationships with the people they communicate with may have reduced mental stress by channeling it in different directions. The development of understanding others and sharing thoughts comfortably may have created a pleasant mood. Experiencing the fulfillment of duties and responsibilities in team sports activities in a game environment may have increased the sense of awareness of their skills, and the variety of cooperation and communication during the game may have contributed to personality development. In the CG, when the pre-test post-test difference is examined, a decreasing significant change was observed mostly in the social benefits dimension, and no significant change was observed in the psychological benefits dimension, which is characterized by the late change process. Although there was no significant change in the CG’s screen viewing time throughout the experimental process, an increase was observed. The significant decrease in the physical and social benefits dimension can be partially explained by the increase in the CG’s screen viewing time.

Khan et al. (2023) examined the relationship between screen time and school performance in a total of 197,439 adolescents (13.6 ± 1.63 years; 51% girls) from Canada and 36 European countries and reported that more than 2 h of screen time per day began to negatively affect school performance. According to the results of the study, whether it is passive (i.e., television) or active (i.e., electronic games, computer use) screen time, as the duration increases, the negative effect on school performance increases in both genders, and it was suggested that reducing screen time will increase school performance.

Studies emphasizing the increase in screen time and decrease in participation in moderate-intensity activities during the pandemic period (Duncan et al., 2023) show that it has significant effects on habit change and habit acquisition, especially in children and adolescents.

The effects of increased screen time and decreased physical and social activities on human behavior, social structure, academic and work life success, and health are undoubtedly among the main problems of countries. In fact, as screen time increases, early effects may start to emerge physically and socially, but the fact that psychological effects are observed less or later does not mean that the problem is insignificant. Although there was no significant difference in the pre-test and post-test change in the psychological sub-dimension score of the LBS in the CG in our research results, the significant increase in the psychological benefit score in the EG reveals that physical activity makes people feel better in every aspect.

Chao (2013) reported a high relationship between leisure time participation, recreation benefit levels and happiness levels of 3,015 secondary school teachers. Koçyiğit et al. (2018) stated that “Socialization through recreation activities directly and significantly affects communication skills.” Chiung et al. (2014) reported that leisure participation has a moderate effect on recreation benefit and motivation. Previous studies, which yielded parallel results to our study, show that people of all age groups and genders who participate in recreative activities and especially physical activities in their free time feel better, which leads to a sound psychological structure and an increase in social communication.

Participation in leisure time activities increases people’s level of happiness (Chao, 2013), develops a sense of self-confidence (Kim et al., 2005), thus physical, psychological and social benefits emerge, and effective use of leisure time leads to leisure time satisfaction (Eskiler et al., 2019; Karoğlu and Atasoy, 2018). In fact, individuals who are aware that they use their free time efficiently are also aware of the benefits of these activities (Ayyıldız and Karaküçük, 2017; Ertüzün et al., 2020).

Although scientific studies have reported different results on the clarity of its effect, studies suggesting that physical activities such as moderate-intensity walking in free time to control hypertension reduce systolic and diastolic blood pressure in hypertensive patients, reveals the importance of free time activities in terms of health (Shariful et al., 2023).

In our study, we observed that participation in activities had a significant positive change in both genders. Although there was no significant difference between genders in the usefulness of participation in activities, male participants showed higher change in terms of physical and psychological benefits, while women showed higher change in terms of social benefits. In the CG, the significant decrease in LBS scores in both genders can be considered as the negative effect of not participating in leisure time activities. This decrease, especially in the physical and social benefit sub-dimensions, shows that men were more affected than women in terms of the social benefit sub-dimension. It is necessary to understand the gender-related effects of leisure activities in more detail and to examine the decreases in the CG in more detail. Some previous studies emphasize similar results of participation in leisure activities in terms of gender (Bülbül et al., 2021; Ertüzün et al., 2020; Karaküçük et al., 2019; Kocaer, 2019). In addition to some studies (Chao, 2013), it has been observed that women have disadvantages (such as time, knowledge and economic status) in participating in recreative activities (Jackson and Henderson, 1995; Bittman and Wajcman, 2000). The results of the studies (Philipp, 1997; Ayhan et al., 2022), in which participation in recreative activities increased and women obtained a higher level of benefit than men, can also be considered in terms of socio-economic status variable or education level.

Previous research results generally show that men’s physical activity participation levels in leisure-time activities are higher than women’s, especially in moderate and high-intensity physical activities. The main reason for this difference is socio-economic level. As the development status of countries and the socio-economic level of individuals increase, leisure-time physical activity participation also increases. Socio-economic deficiency is based on both the lack of physical activity opportunities and the lack of information about leisure benefits. Another possibility may be due to the increased time spent in working life in low-income countries. In addition, when we look at the relationship between physical activity, gender and age, it is seen that physical activity participation decreases as age increases, more so in women (Azevedo et al., 2007).

The fact that there was no significant difference in our study in terms of age variable can be explained by the homogeneity of the age distribution of the participant group. These results suggest that the benefits of leisure activities of people in similar age groups have similar effects.

Physical activity contributes positively to life satisfaction, while personal internet use and TV consumption negatively affect life contentment and life satisfaction (Schmiedeberg and Schröder, 2017; Frey et al., 2019; Leung and Lee, 2005). In addition, based on the fact that those who do not have internet access are happier (Cuñado and de Gracia, 2012), it is necessary to understand the importance of recreative activities, especially those involving physical activity.

Our suggestion to other researchers is that supporting this importance with longitudinal and experimental studies and including demographic characteristics such as economic conditions, social status, welfare level, family education level, etc. in the study will further increase the importance of this study. If we touch on the weaknesses of the research, although there were 176 participants in the study, the fact that the participants came from a single field of expertise (mathematics) and a single province limits the generalization of the results. Expanding the research to include students in other fields and universities may yield more relevant and broadly applicable results.

According to our study results, it is very important to increase the habit and incentive to participate in leisure activities during the university student period, which is the most productive period of young people, in order to distract them from screen addiction and to ensure well-being fitness. Recreative activities are seen as an important tool to intervene in screen time. In addition to the physical and social effects that may show early symptoms, encouraging participation in activities is a very effective solution tool in order to prioritize the negativities of the psychological effects that occur later. It is predicted that if recreational activities, especially activities involving physical activity, are not made a habit at the ages in which the study is concerned, the individual will become more physically and psychologically worn out in the following years.

## Data Availability

The data supporting the conclusions of this article will be made available by the authors upon request. Requests to access the datasets should be directed to olcaymulazimoglu@mu.edu.tr.
